# Neural Synchronization, Chimera States and Sleep Asymmetry

**DOI:** 10.3389/fnetp.2021.734332

**Published:** 2021-10-12

**Authors:** Tera A. Glaze, Sonya Bahar

**Affiliations:** Department of Physics and Astronomy and Center for Neurodynamics, University of Missouri at St. Louis, St. Louis, MO, United States

**Keywords:** chimera states, neural synchronization, sleep dynamics, unihemispheric sleep, asymmetric sleep

## Abstract

We model the dynamics of sleep states in two connected model brain hemispheres, using groups of coupled individual Hindmarsh-Rose neural oscillators. In a single isloated hemisphere, sleep-promoting neurons and wake-promoting neurons exhibit alternating levels of within-group mean field activity, as well as alternating levels of stochastic phase synchronization, as the system moves between simulated day and night. In a two-hemisphere model, we find differences in the behavior of the sleep-promototing or wake-promoting regions between hemispheres, indicative of chimera-like behavior. We observe phase-cluster states, in which different hemispheres exhibit different bursting dynamics, as well as differences in synchronization between hemispheres. This provides a basis for modeling unihemispheric sleep, which occurs naturally in cetaceans and some bird species, among others, as well as asymmetric sleep, which occurs in human subjects suffering from sleep apnea or experiencing the “first night effect” induced by sleeping in a novel environment.

## Introduction

Sleep is a nearly ubiquitous phenomenon among living organisms. Even the jellyfish *Cassiopea*, which lacks a centralized nervous system, exhibits a sleep-like state (Nath et al., 2017). Yet the reason for the necessity of sleep, and the processes that control it, are far from well understood, despite a rapidly growing literature on the genetic regulation of sleep and other circadian rhythms in animals (Rijo-Ferreira and Takahashi, 2019) and plants (Oakenfull and Davis, 2017; Creux and Harmer, 2019), and extensive studies of the neural regions controlling sleep states in mammals (Scammell et al., 2017).

Sleep is an inherently dynamical phenomenon. In the mammalian brain, sleep is modulated by external drives such as the light-dark cycle, and also by an internal circadian rhythm generated within the suprachiasmatic nucleus (SCN) (Moore and Eichler, 1972), which receives input from external light stimuli (Moore and Lenn, 1972). In concert with external light stimuli, the SCN moderates a mammal’s daily and seasonal rhythms and behaviors (Aton and Herzog, 2005). Computational studies of sleep dynamics often use empirically-based models of the generated circadian rhythm, rather than simulating neural activity in the SCN. These include the model of Daan et al. (1984), who used a skewed sine wave as the circadian drive; a two-oscillator model developed by Strogatz (1987); a square array of SCN oscillators (Kunz and Achermann, 2003); and a light-based model with an additional non-photic input (St. Hilaire et al., 2007).

From a dynamical standpoint, sleep can be pictured as a phenomenon of neural synchronization modulated by internal pacemakers in the SCN and external drives such as the light-dark cycle. However, the situation is significantly complicated by the fact that various brain regions are involved in mutual excitatory and inhibitory interactions which regulate sleep processes, as will be discussed further below. Moreover, brains have two hemispheres, and sleep is not always symmetric.

Most mammals experience bihemispheric sleep (BHS), in which both hemispheres exhibit the same sleep state at the same time (Rattenborg et al., 2000; Corsi-Cabrera et al., 2006). Although not a very common occurrence, interhemispheric asymmetry has been observed in human sleep (Braun et al., 1997). Asymmetry can arise from separation of the hemispheres through surgery (Corsi-Cabrera et al., 2006), and also in humans with sleep apnea (Abeyratne et al., 2010; Rial et al., 2013), which has been found to correlate with the magnitude of hemispheric asymmetry (Abeyratne et al., 2010). During normal breathing in sleep, apneic patients exhibit asymmetry; at the onset of an apneic episode, the hemispheres resynchronize (Rial et al., 2013).

Asymmetry between hemispheres during sleep can also occur in healthy humans, as discovered by Tamaki et al. (2016). When humans fall asleep in a new, unfamiliar location, portions of one hemisphere do not sleep as deeply as the other hemisphere, maintaining a heightened awareness of the environment. During this time, unfamiliar sounds will arouse a person more frequently and with faster response time when detected by the more lightly sleeping hemisphere than when detected by the more deeply sleeping hemisphere. This phenomenon has only been observed during the first night in a novel environment and is thus called the “first night effect”.

Unlike humans, during normal sleep Cetaceans (whales, dolphins and porpoises) allow one hemisphere at a time to sleep while the other maintains vigilance, switching multiple times during a period of rest. This form of sleep is called unihemispheric sleep (UHS), characterized by one hemisphere exhibiting an electroencephalography (EEG) pattern congruent with non-rapid eye movement (NREM) sleep (characterized by high amplitude and low frequency, synchronized), while the other hemisphere shows an EEG pattern that indicates wakefulness (low amplitude and high frequency, desynchronized). The wakeful hemisphere can exhibit intermediate activity between NREM and wakefulness, without dipping so far into sleep that both hemispheres are considered in the same state (Rattenborg et al., 2000). An early EEG study by Mukhametov et al. (1977) found simultaneous, independent synchronization and desynchronization in the two hemispheres of the dolphin brain.

Even before the discovery of UHS, some birds’ ability to fly continuously for days at a time was a scientific puzzle. When did they sleep? Due to the size mismatch between tiny avian subjects and large experimental recording apparatus, studies have been limited (Rattenborg et al., 2000; Rattenborg, 2017). Scientists hypothesized, based on visual observations and indirect studies, that birds might fly either using only one hemisphere (UHS), or simply lock their wings and glide (BHS), supported by the evidence that birds are still capable of flight after the connections between the brain and the spinal cord had been severed (Rattenborg et al., 2000). Indeed, due to newer tracking capabilities, it has been found that great frigatebirds (*Fregata minor*) do utilize both UHS and BHS while they fly. However, the amount of time they spend sleeping during flight was surprisingly small, less than an hour per day (mostly UHS or asymmetric sleep), in contrast to nearly 13 h of sleep per day while nesting (Rattenborg, 2017).

In contrast to studies of birds in flight, birds exhibit UHS conditionally while resting on land. Rattenborg et al. (1999) studied Mallard ducks (*Anas platyrhynchos*) and showed that, when sleeping in groups, the ducks showed a predilection for sleeping unihemispherically when on the outer edge of the group, with the open eye facing away from the group, presumably to watch for predators. Ducks in the center showed no preference for which eye they held open during UHS, and also exhibited less UHS than those on the outer edge (Rattenborg et al., 1999). Some species adjust their behavior from UHS to BHS depending on circumstances. For example, eared seals experience UHS while in the water and BHS on land. In the water, they use their “awake” hemisphere to paddle and keep their face above water to breathe, occasionally switching sides (Rattenborg et al., 2000). Rapid eye movement (REM) sleep is not present during UHS; it has been suggested that REM has been lost in aquatic mammals due to natural selection in response to predators or other environmental pressures, the need to remain at or regularly return to the surface for air, and/or temperature maintenance (Madan and Jha, 2012). Consistent with this hypothesis, fur seals have been recently shown to suppress REM sleep for extended periods of time (up to 2 weeks) while in the water (Lyamin et al., 2018).

Many researchers have suggested an analogy between unihemispheric or asymmetric sleep and chimera states (Abrams et al., 2008; Tinsley et al., 2012; Panaggio and Abrams, 2015; Majhi et al., 2019; Wang and Liu, 2020). A chimera state is a dynamical state in which subsets of an ensemble of identical, interacting oscillators exhibit distinct dynamical states, such as one group of synchronized oscillators and one group of desynchronized oscillators (Abrams and Strogatz, 2004). Chimera states have been found in systems of different types of oscillators, including mechanical (Martens et al., 2013), optical (Hagerstrom et al., 2012), chemical (Tinsley et al., 2012; Nkomo et al., 2013; Wickramasinghe and Kiss, 2013), and of course neural (Omelchenko et al., 2013, Hizanidis et al., 2014; Glaze et al., 2016, and others; see Majhi et al., 2019 for review). Systems that generate chimera states can also exhibit phase-cluster states, in which different groups exhibit different synchronized oscillatory patterns (Tinsley et al., 2012). In the present paper, we develop a model of unihemispheric sleep incorporating individual neural oscillators. Unihemispheric sleep was modeled by Kedziora et al. (2012), who adapted a preexisting model to create two hemispheres, which alternately switched between sleep and wake states. We take inspiration from this approach, but develop a model based on coupled individual neurons, rather than single equations governing entire regions of the brain. This approach is novel in that it allows for the examination of interactions not only between regions, as can be done with neuronal mass models, but also within regions, using measures such as stochastic phase synchronization (Pikovsky et al., 2001). As we will show below, asymmetric sleep dynamics are observed in the model, in the form of chimera-like dynamical states, and alternations between levels of synchronization are observed within the sleep-promoting and wake-promoting neural regions throughout the simulated circadian cycle.

## Model Background and Design

The simplest form of a sleep-wake model is a “flip-flop” switch based on the interaction between neurons that promote a sleep state (such as those in the ventrolateral preoptic area, or VLPO), and neurons that promote a wake state (such as neurons in the locus coeruleus, or LC). In such models, each state is stable on its own, but an external drive (such as homeostatic sleep pressure) and mutual inhibition between the two groups cause the overall system state to switch from wake to sleep or vice versa (Gallopin et al., 2000; McGinty and Szymusiak, 2000; Saper et al., 2001; Nakao et al., 2007; Rempe et al., 2010). Booth and Diniz Behn (2014) developed a flip-flop-like model that exhibited hysteresis as the external drive was tuned. They showed that their results were comparable to the two-process model developed by Daan et al. (1984), which incorporates two separate, interacting processes corresponding to the circadian drive or rhythm, and the sleep propensity, or the homeostatic drive. These approaches were used by Kedziora et al. (2012) in order to investigate unihemispheric sleep in a two-hemisphere neuronal mass model.

**FIGURE 1:**
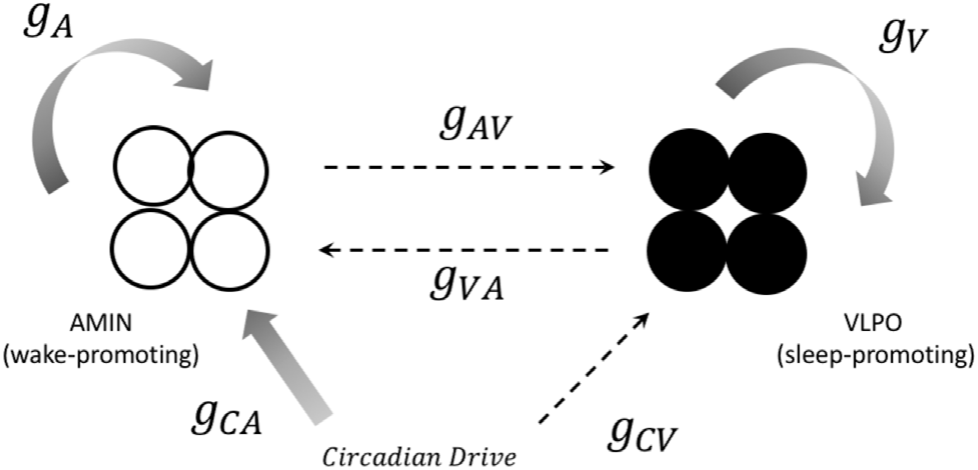
A representation of the connections between components in the one-hemisphere model. Both the sleep (black circles) and wake (open circles) regions consist of four neurons. Solid arrows represent excitatory projections, and dashed arrows represent inhibitory projections. The notation for the corresponding coupling constant is shown next to each arrow. See text for more details.

The model used in the present paper combines aspects of these approaches with dynamical models of individual neural oscillators. This allows for the comparison of neural synchronization within subpopulations of oscillators, rather than simply comparisons between brain regions, as in the neural mass models such as those developed by Kedziora et al. (2012). For each “hemisphere,” we consider a small group of neurons **(**typically four neurons, unless otherwise specified) that are active during the wake state (corresponding to AMIN neurons in the locus coeruleus), another group of neurons active during the sleep state (corresponding to the VLPO region), and a circadian pacemaker which drives the state-switching. The sleep and wake groups mutually inhibit each other, and the state of the system is determined by the (more) active group. A schematic diagram for one hemisphere is shown in Figure 1. This can be compared to the approach of Postnova et al. (2009), who modeled sleep-wake cycles based on feedback between two individual neurons.

The role of the ventrolateral preoptic area (VLPO) in sleep regulation was first recognized with the demonstration of insomnia in rats whose hypothalamic preoptic area had been lesioned (Nauta, 1946). That the VLPO specifically contains sleep-promoting neurons was not discovered, however, until 1996 (Sherin et al., 1996). A reciprocal inhibitory relationship has been observed between the VLPO and the wake-promoting regions of the hypothalamus, leading to the use of the VLPO in flip-flop switch models (Gallopin et al., 2000; McGinty and Szymusiak, 2000; Saper et al., 2001; Saper and Lowell, 2014). VLPO activity has also been simulated in more complex models of sleep-wake dynamics, including that of Phillips and Robinson (2007), a model developed to replicate mouse sleep-wake behavior (Diniz Behn et al., 2007), the two-hemisphere sleep-wake model developed by Kedziora et al. (2012) to simulate unihemispheric sleep, and others. In the present work, we will associate the sleep-promoting neurons with the VLPO region.

Monoaminergic neurons (typically referred to as AMIN neurons) in the locus coeruleus have been shown to promote wakefulness (Diniz Behn et al., 2007). The LC and VLPO have reciprocal inhibitory connections (Saper et al., 2001; Saper et al., 2010), making AMIN neurons a prime choice to pair with the VLPO for flip-flop switch models. AMIN neurons from the LC are also frequently used in other sleep models to represent a group or region that promotes waking (Diniz Behn et al., 2007; Phillips and Robinson 2007). In the present model, we will associate the wake-promoting neurons with AMIN neurons in the locus coeruleus.

In the model used here, the circadian pacemaker is a skewed sine wave with its peak in the early day and the trough occurring in early night, as defined by Daan et al. (1984). The input from the pacemaker function is given as

$$I_{c}=0.97\sin\left(\omega t\right)+0.22\sin\left(2\omega t\right)+\;0.07\sin\left(3\omega t\right)+0.03\sin\left(4\omega t\right)+\;0.01\sin\left(5\omega t\right)$$ (1)

It has a range from −1 to 1, with ω=2π/T , where the period *T* is the length of a full day (Daan et al., 1984). This function is interpreted as combining the internal action of the SCN and the external drive from the light/dark cycle.

Individual neurons are modeled using the three-dimensional version of the Hindmarsh-Rose model (Hindmarsh and Rose, 1984), which consists of three coupled nonlinear differential equations:

$$\dot{x}=y-ax^{3}+bx^{2}+I-z\;-\xi +C_{i}$$ (2a)

$$\dot{y}=c-\text{d}x^{2}-y$$ (2b)

$$\dot{z}=r\left(s\left(x-x_{1}\right)-z\right)$$ (2c)

Here, x is the membrane potential or voltage of the neuron, y is the recovery variable, and z is the adaptation current. I is the applied or external current and controls the bursting behavior of the neuron. Unless otherwise noted, parameters are set as a=1,  c=1,  d=5,  r=0.003,  s=4 , and $x_{1}=\;-1.6$ . The parameter ξ represents a Gaussian white noise term, generated using the Fox et al. (1988) algorithm as implemented by Braun et al. (1998), with *D* = 0.005. The model takes on a range of natural frequencies depending on the parameters used. As the current *I* is tuned, uncoupled Hindmarsh-Rose neurons undergo a transition from single spikes to bursting and chaotic dynamics (González-Miranda, 2007). In the single-spiking regime, for example, an uncoupled Hindmarsh-Rose neuron will fire one spike every ∼200–400 time units, which are usually treated as milliseconds in order to align with a typical neural firing timescale, depending on the value of *I*.

Wake-promoting region parameters are designated with the subscript *A* (for AMIN), and sleep-promoting region parameters are designated with the subscript *V* (for VLPO). Each neuron receives input from all other neurons as well as the circadian drive. These inputs are combined in the coupling term $C_{i}$ , giving, for the i th AMIN neuron,

$$C_{i}=g_{A}{\bar{V}}_{Ai}+g_{VA}{\bar{V}}_{V}\;+g_{CA}I_{C}$$ (3)

where $g_{A}$ represents the coupling coefficient among the AMIN neurons, ${\bar{V}}_{Ai}$ is

$${\bar{V}}_{Ai}=\left[V_{i}\left(t\right)-\frac{{\text{Σ}}_{i\neq j}V_{j}\left(t-\tau \right)}{N_{neur}-1}\right]$$ (4)

with $V_{i}\left(t\right)$ corresponding to the x-coordinate of the neuron of interest, and $N_{neur}$ the number of neurons in the AMIN group, with summation over all neurons in the AMIN group except the neuron of interest. The coefficient $g_{VA}$ corresponds to the coupling strength from the VLPO to the AMIN region, with

$${\bar{V}}_{V}=\left[V_{i}\left(t\right)-\frac{{\text{Σ}}_{j}V_{j}\left(t-\tau \right)}{N_{neur}}\right]$$ (5)

where the second term in the brackets corresponds to the mean field of the VLPO region. Lastly, the coefficient $g_{CA}$ gives the coupling strength of the circadian drive (1) to the neuron of interest. For the sleep-promoting VLPO neurons, analogous equations are used, with

$$C_{i}=g_{V}{\bar{V}}_{Vi}+g_{AV}{\bar{V}}_{A}\;+g_{CV}I_{C}$$ (6)

$${\bar{V}}_{Vi}=\left[V_{i}\left(t\right)-\frac{{\text{Σ}}_{i\neq j}V_{j}\left(t-\tau \right)}{N_{neur}-1}\right]$$ (7)

$$\;{\bar{V}}_{A}=\left[V_{i}\left(t\right)-\frac{{\text{Σ}}_{j}V_{j}\left(t-\tau \right)}{N_{neur}}\right]$$ (8)

and summation in Eq. 7 over the VLPO neurons except the neuron of interest, and the summation in Eq. 8 over the AMIN neurons.

To make the wake neurons active during the day, at the peak of the circadian drive (CD), and inactive during the night, at the trough of CD, the projection from CD to the AMIN region is excitatory, and the projection from CD to the VLPO region is inhibitory. The time delay τ corresponds to the finite time needed for signal transmission. This delay is shorter for the neurons within a region and is longer between regions. These delays are set as τ=10.40 ms for neurons within one group, and τ=21.00 ms between neurons in different groups. The model is implemented using a custom-written MATLAB code, using Euler integration with a step size of dt=0.01 ms. The simulation is started from heterogeneous initial conditions, with values of the “voltage” variable *x* uniformly distributed between −2 and 2, and the *y* and *z* variables initialized to zero.

Synchronization within and between groups is assessed using stochastic phase synchronization analysis. Briefly, two oscillators are considered synchronized if their phase difference ϕ remains relatively constant over time. The 1:1 phase difference between two neurons, *i* and *k*, is defined as

$$\phi_{ik}\left(t_{i}\right)=2\pi \left(t_{i}-t_{k}\right)/\left(t_{k+1}-t_{k}\right)$$ (9)

where neuron *i* spikes at time $t_{i}$ , while $t_{k}$ and $t_{k+1}$ are two sequential spike times for neuron k , and $t_{k}<t_{i}<t_{k+1}$ . The more ϕ changes, the less synchronized the neurons are. Synchronization can be quantified using the synchronization index

$$\gamma_{ik}^{2}=\langle \cos{\langle \phi_{ik}\left(t_{i}\right)\rangle }^{2}+\sin{\langle \phi_{ik}\left(t_{i}\right)\rangle \rangle }^{2}$$ (10)

where the brackets denote time averages. This corresponds to the intensity of the first Fourier mode of the distribution of phase differences. If γ is equal to 1, the oscillators are perfectly synchronized, while if γ=0 , they are completely desynchronized (Pikovsky et al., 2001).

## Results

The Hindmarsh-Rose model exhibits different bursting states as the parameter I is tuned in Eq. 2a (González-Miranda, 2007). We investigated the dynamics of the single hemisphere model described above and shown schematically in Figure 1, for values of I in both the single-spiking and the bursting regimes. All other parameters were held constant at $g_{A}=g_{V}=0.000045$ , $g_{AV}=-7.5x10^{-6}$ , $g_{VA}=-4.25x10^{-5}$ , I=1.28 , $g_{CA}=1.15x10^{-3}$ , $g_{CV}=-0.0019$ . Parameters are identical for the VLPO and AMIN neurons; they are differentiated only by their interactions with the circadian drive.

For the case in which uncoupled neurons exhibit single spikies ( I=1.28 ), the AMIN neurons were active during simulated “day” (defined by the positive half-cycle of the circadian oscillation) while the VLPO neurons were completely inactive. During the simulated “night” (defined by the negative half-cycle of the circadian oscillation), the reverse was observed: the VLPO neurons were active, while the AMIN neurons were completely inactive. For parameters for which uncoupled neurons exhibit bursting dynamics ( I=1.75, I=2.0 ), both the AMIN and VLPO neurons remained active during both the simulated “day” and “night.” However, the average activity (mean field) of AMIN was greater than that of VLPO at the peak of the circadian drive cycle, while the VLPO mean field was greater during the simulated night.

**FIGURE 2:**
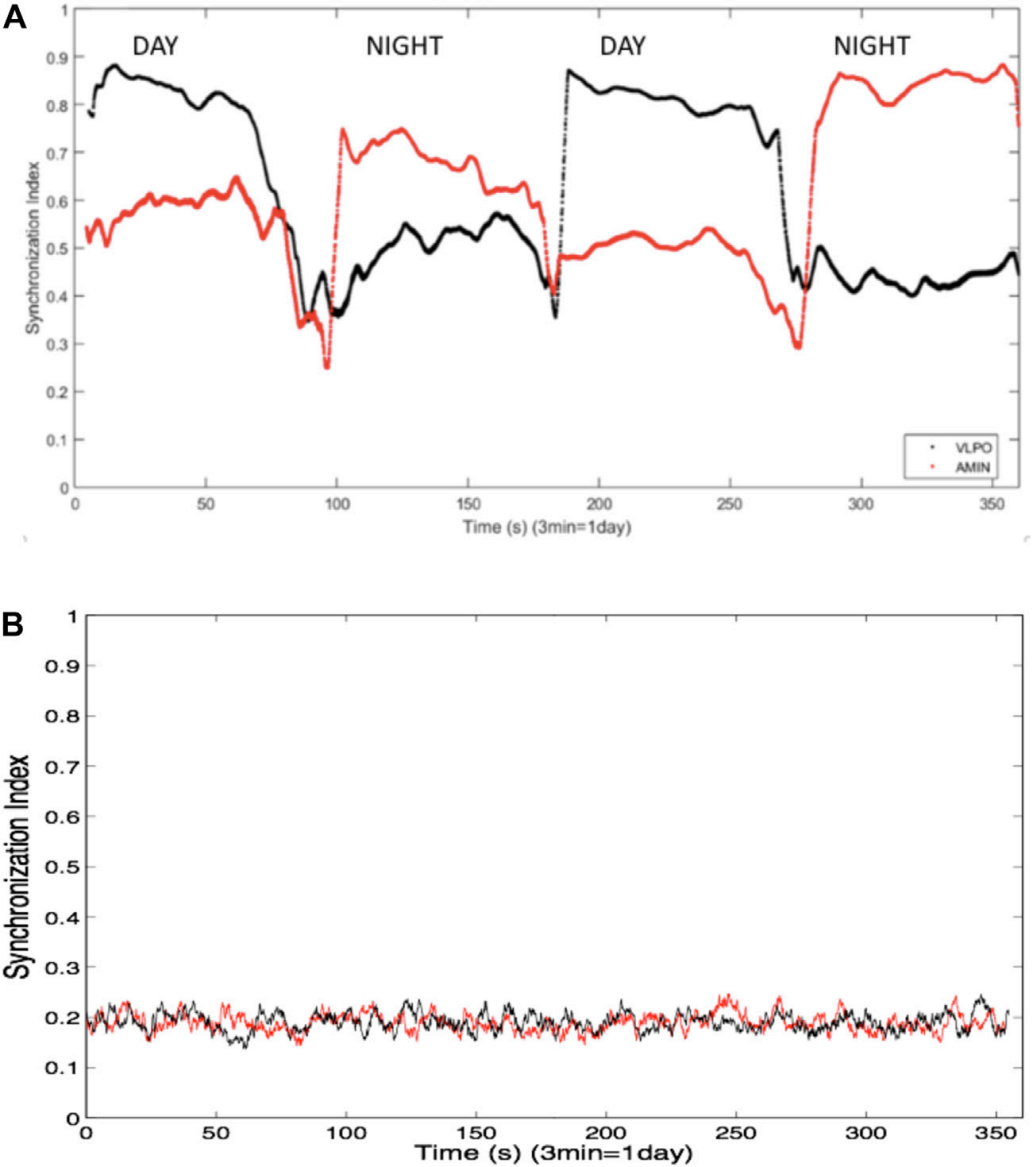
**(A)** Synchronization indices averaged over all non-identical pairs of neurons the VLPO region (black trace) and the AMIN region (red trace) in the single hemisphere model using a sliding window of 100 spikes, and over ten replicate data sets. Parameters are $g_{A}=g_{V}=0.000045$ , $g_{AV}=-7.5x10^{-6}$ , $g_{VA}=-4.25x10^{-5}$ , I=2.00 , $g_{CA}=1.15x10^{-3}$ , $g_{CV}=-0.0019$ . Three minutes of simulated time corresponds to one “day”, in which the circadian drive (not shown) completes a full cycle. **(B)** Synchronization indices for a shuffled data set, calculated using a sliding window of 100 spikes, for VLPO (black trace) and AMIN (red trace).

Synchronization within the AMIN and VLPO groups was assessed using phase synchronization analysis, as described in the previous section, with a sliding window 100 spikes wide and a step forward of one spike, allowing for the analysis of the synchronization index as a function of time. In the bursting regime, greater synchronization was observed in the AMIN neurons during the night, and greater synchronization in the VLPO neurons during the day. In other words, synchronization correlated inversely with overall activity, as shown in Figure 2A, for I=2.0 . Synchronization indices are averaged over all non-identical pairs of neurons in each region, and over ten replicate data sets. (Note that synchronization could not be assessed in the single-spiking regime during the night for AMIN or the day for VLPO, since those regions were entirely quiescent during those intervals, and thus comparison of day/night synchrony is only possible for the bursting regime.)

In order to confirm that the ordering of the spike trains was responsible for the synchronization, the spike times were shuffled while retaining the distribution of interspike intervals. Figure 2B shows the averaged synchronization index, again using a 100-spike sliding window, between the shuffled spike trains from all non-identical neuron pairs for one of the ten data sets. The synchronization is markedly decreased in comparison to the indices shown in Figure 2A, and no difference is observed between the indices for the AMIN and VLPO neural pairs. Similar results are obtained upon shuffling the other data sets used to generate Figure 2A.

**FIGURE 3:**
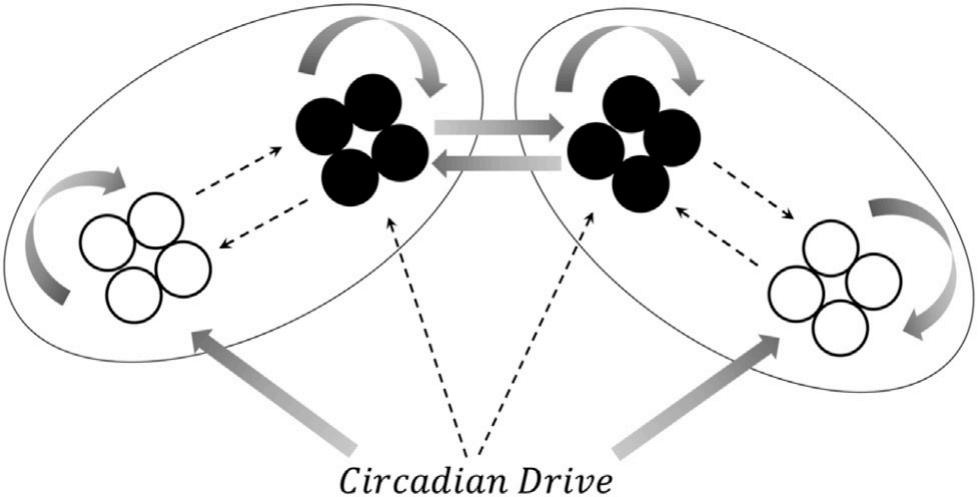
A schematic representation of the two-hemisphere version of the model. Connections within each hemisphere are identical those shown in Figure 1, though the circadian drive now projects to the AMIN and VLPO regions in each hemisphere. Additionally, excitatory connections are added between the right and left VLPO regions. See text for details.

The single hemisphere model shown in Figure 1 can be extended to a two-hemisphere model, shown schematically in Figure 3. Each hemisphere has its own VLPO and AMIN regions, each consisting of a group of individual neurons. The circadian drive projects to each of the VLPO and AMIN regions in the same fashion as the single-hemisphere model. The hemispheres communicate *via* excitatory connections (solid arrows) between the VLPO regions. This form of the model was inspired by the two-hemisphere sleep-wake model designed by Kedziora et al. (2012). With the exception of the added excitatory coupling between VLPO regions, the parameters in the two-hemisphere model use the same naming scheme as in Figure 1. For the new cross-hemispheric VLPO connections, the coupling constants are given as $g_{LR}$ (left VLPO to right VLPO) and $g_{RL}$ (right VLPO to left VLPO). Parameters are identical for both hemispheres, and the interhemispheric coupling is symmetric ( $g_{LR}=g_{RL}$ ). The terms “left” and “right” are arbitrary designations for the hemispheres; there is no explicit spatial orientation in the model.

**FIGURE 4:**
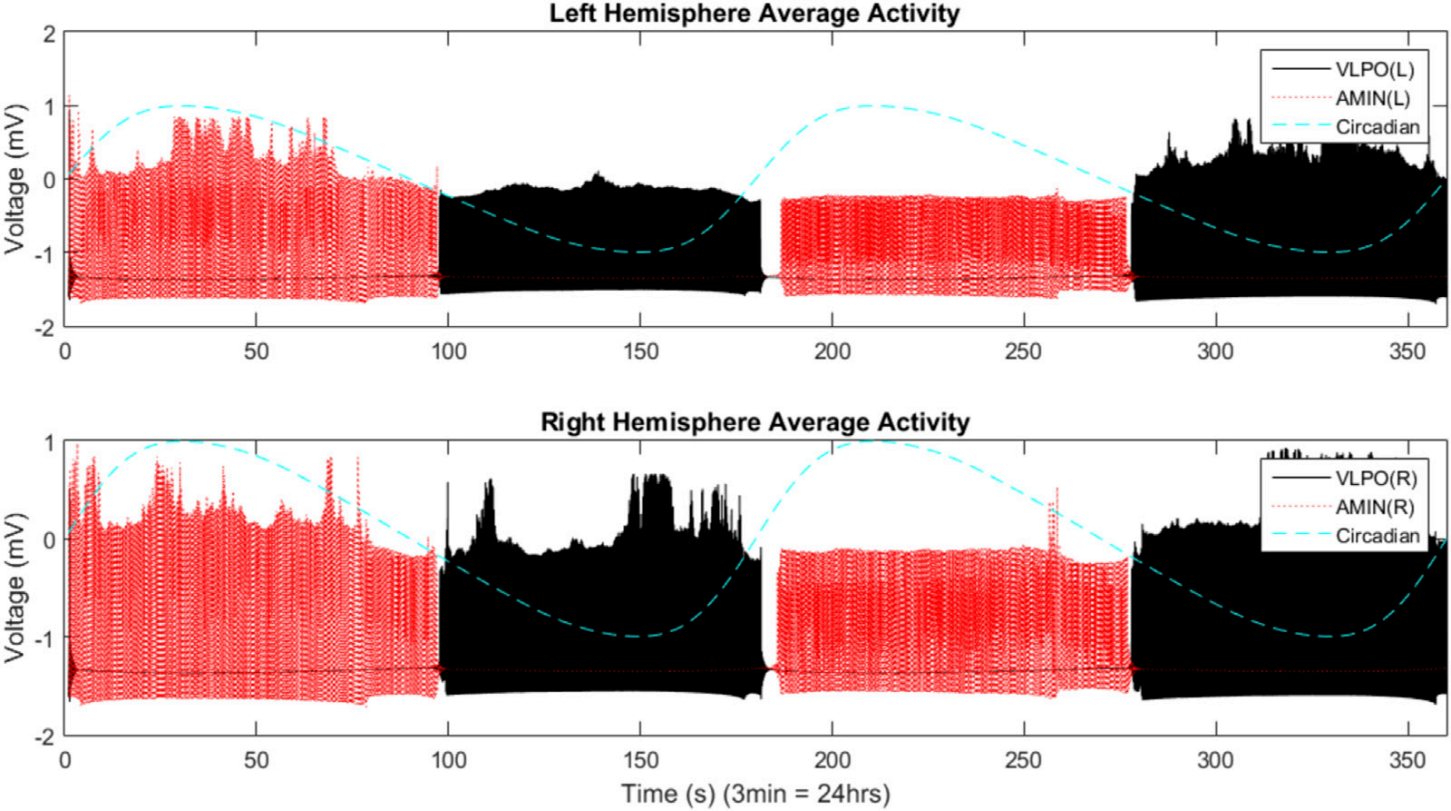
Mean field activity in the two-hemisphere model in the regime where uncoupled neurons fire single spikes. The mean field is calculated as the average value of the x variable over all neurons in any give group at each time point. The vertical axis is labeled with “voltage” in units of “mV” to reflect the fact that this variable is analogous to the transmembrane potential. The mean field activity of the “left” hemisphere is shown in the top panel, and that of the “right” hemisphere in the bottom panel. AMIN activity is shown in red, and VLPO is shown in black. The circadian oscillations given in Eq. 1 are shown with the blue dashed line. Parameters are $g_{A}=g_{V}=0.000045$ , $g_{AV}=-0.0000075$ , $g_{VA}=-0.0000425$ , $g_{CA}=0.00115$ , $g_{CV}=-0.0019$ , $g_{LR}=g_{RL}=0.00002$ , and I=1.295 , with four neurons per region.

The two-hemisphere model can generate chimera states in which the hemispheres exhibit significantly different dynamical behaviors. The mean field activity for each hemisphere, when the system is in the single-spiking regime ( I=1.295 ), is shown in Figure 4. Here, as for the single-hemisphere single-spiking regime, the wake-promoting AMIN region is only active during the day, while the sleep-promoting region VLPO is only active during the night. Even though the parameters of each hemisphere are set identically, the hemispheres exhibit independent variations in mean field activity. The right VLPO, for example, is much more active during the first night than the left. Variations in the synchronization indices between the active regions in the two hemispheres are also observed (data not shown).

**FIGURE 5:**
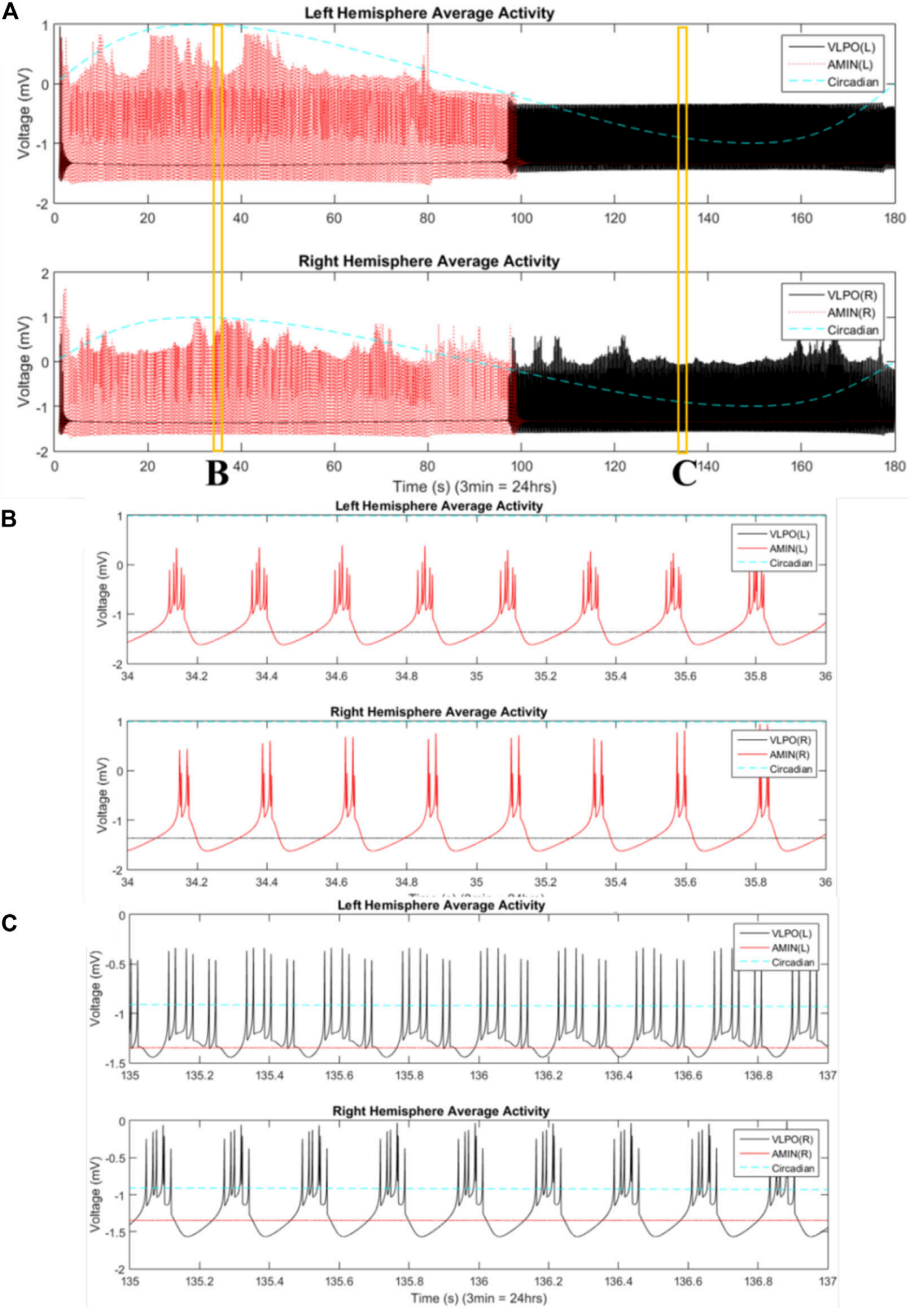
Phase-cluster chimera states are observed between hemispheres. **(A)** Mean field activity of the “left” hemisphere is shown in the top panel, and that of the “right” hemisphere in the bottom panel. AMIN activity is shown in red, and VLPO is shown in black. The circadian oscillations given in Eq. 1 are shown with the blue dashed line. Parameters are $g_{A}=g_{V}=0.000045$ , $g_{AV}=-0.0000075$ , $g_{VA}=-0.0000425$ , $g_{CA}=0.00115$ , $g_{CV}=-0.0019$ , $g_{LR}=g_{RL}=0.00002$ , and I=1.3 , with 3 neurons per region. **(B)** Magnification of a time interval from panel **(A)**, 34–36s. **(C)** Magnification of panel A, 135–137s.

The mean field activity of the VLPO and AMIN for I=1.30 , still in the single-spiking regime, is shown in Figure 5. Interhemispheric asymmetries are evident, especially for the VLPO region at night. Magnification of representative mean field dynamics for each region is shown in Figures 5B,C. In Figure 5B, the left hemisphere AMIN exhibits tight clusters of multi-spike bursts, while the right hemisphere shows double spikes, indicating that all the neurons are simultaneously firing doublets. This difference in behavior between coupled identical groups is characteristic of a phase-cluster chimera state (Tinsley et al., 2012). In Figure 5C, the VLPO also exhibits a phase-cluster chimera state, with tight, clustered firing in the right hemisphere, and nearly evenly-spaced cascades of spike pairs in the left hemisphere.

**FIGURE 6:**
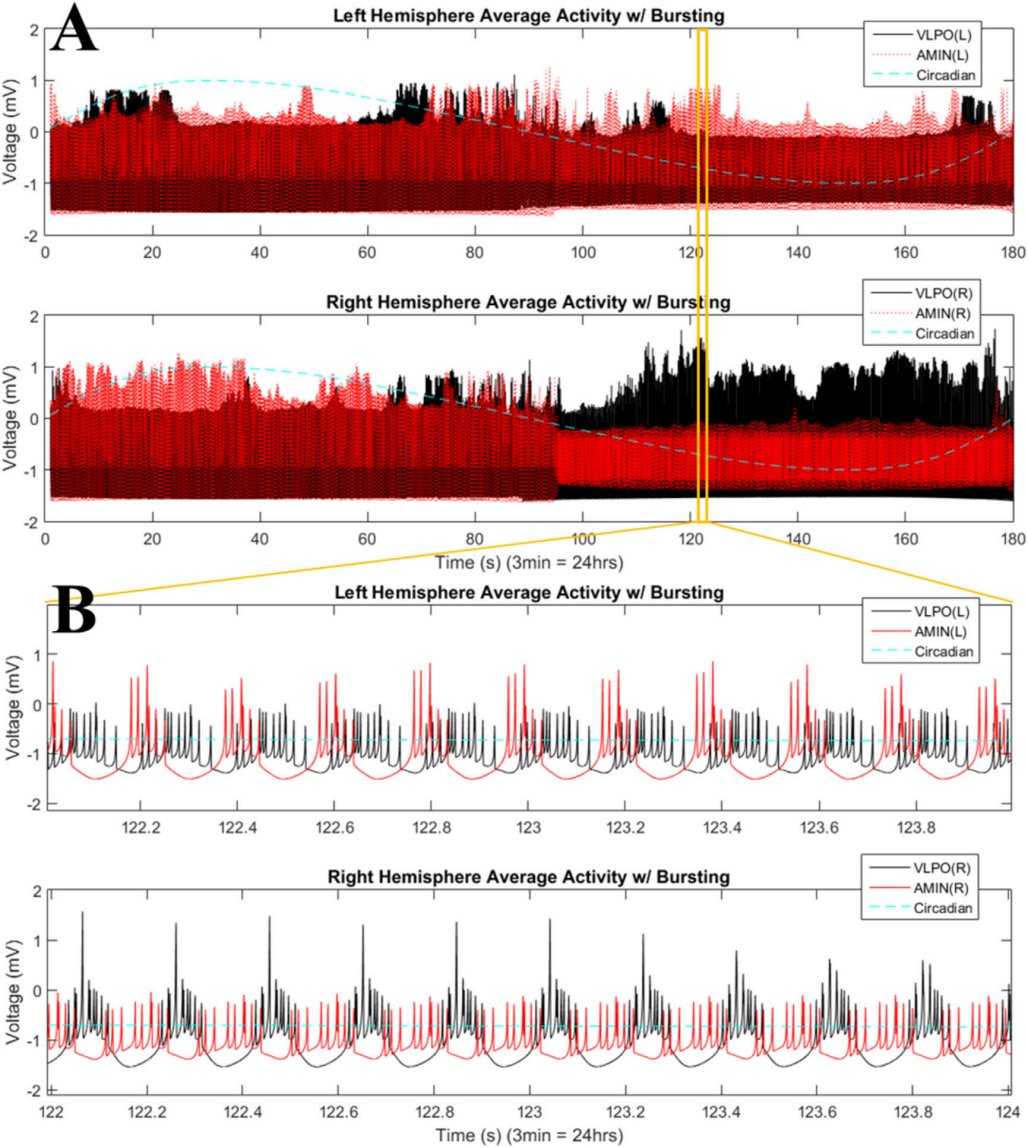
Phase-cluster states in the bursting regime for the two-hemisphere model. **(A)** Mean field activity is shown for the left (top panel) and right (bottom panel) hemispheres. AMIN activity is shown in red, and VLPO is shown in black. The circadian oscillations given in Eq. 1 are shown with the blue dashed line. **(B)** Magnification of a time interval from **(A)**, 122–124s, with the top panel showing mean field activity from the left hemisphere and the lower panel showing mean field activity from the right hemisphere. Parameters are $g_{A}=g_{V}=0.000045$ , $g_{AV}=-0.0000075$ , $g_{VA}=-0.0000425,\;\;g_{CA}=0.00115$ , $g_{CV}=-0.0019$ , $g_{LR}=g_{RL}=0.00002$ , and I=2.00 , with 3 neurons per region.

Moving into the bursting regime, with I=2.00 , the neurons of all regions fire over the entire day, as in the single-hemisphere simulations, though there is interhemispheric asymmetry in the activity level (Figure 6A). Zooming in on an interval of daytime activity (from 122 to 124 s) shows tight, clustered firing for AMIN in the left and VLPO in the right hemisphere, and a cascading firing pattern for VLPO in the left and AMIN in the right hemisphere (6B). This different behavior for both regions across hemispheres is again evidence of a phase-cluster chimera state. As with many other studies of chimera-like behavior (Wolfrum and Omel’chenko, 2011), this phase-cluster state is transient, and does not persist throughout the entire duration of the simulation. We note that the frequency of the mean field oscillations of the coupled system (see Figures 5B,C, 6C) will not be a simple average of the frequencies of the individual oscillators; see Petkoski et al. (2013) for a detailed investigation of how the mean field of a system of coupled oscillators relates to the individual oscillator frequencies.

**FIGURE 7:**
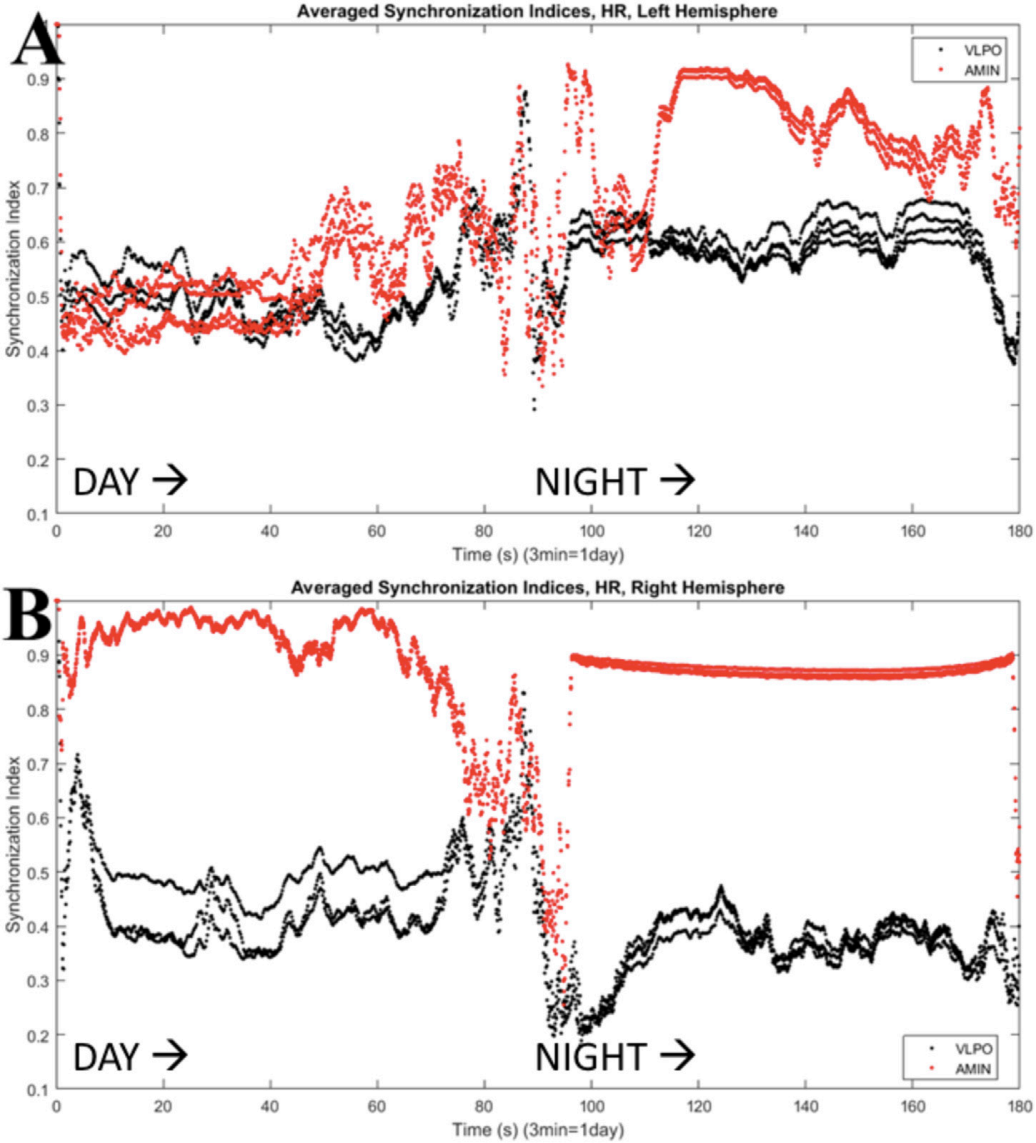
Synchronization indices calculated over a 10-spike sliding window for each hemisphere, with a one-spike step forward, from data shown in Figure 6. AMIN synchronization indices are shown in red, and VLPO is shown in black. **(A)** Left hemisphere synchronization. **(B)** Right hemisphere synchronization. Parameters are $g_{A}=g_{V}=0.000045$ , $g_{AV}=-0.0000075$ , $g_{VA}=-0.0000425,\;\;g_{CA}=0.00115$ *,* $g_{CV}=-0.0019$ , $g_{LR}=g_{RL}=0.00002$ , and I=2.00 , with 3 neurons per region. The multiple lines in the synchronization index are a result of averaging over a short time window in the presence of burst-firing.

**FIGURE 8:**
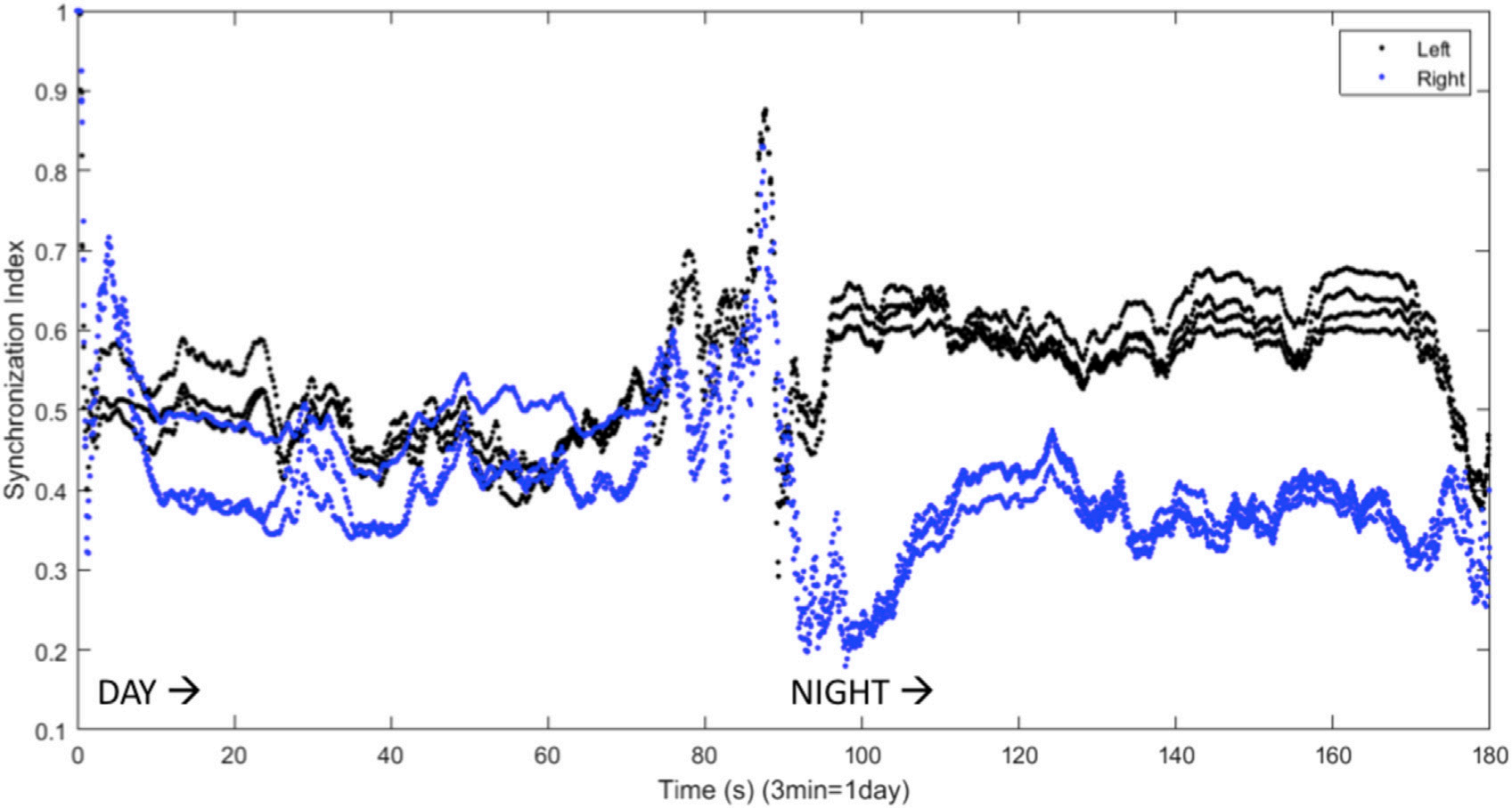
Synchronization indices calculated over a 10-spike sliding window for the left and right VLPO, with a one-spike step forward, from data shown in Figures 6, 7. This figure combines the VLPO synchronization indices from Figure 7 for ease of visual comparison. The left hemisphere VLPO is shown with the black trace, and the right with the blue trace. Parameters are $g_{A}=g_{V}=0.000045$ , $g_{AV}=-0.0000075$ , $g_{VA}=-0.0000425,\;\;g_{CA}=0.00115$ , $g_{CV}=-0.0019$ , $g_{LR}=g_{RL}=0.00002$ , and I=2.00 , with 3 neurons per region. Note the difference in synchronization indices during the second half of the time series (simulated “night”).

The results shown in Figure 6 can be quantified using the synchronization index, which reveals significantly different levels of synchronization between the left and right hemispheres (Figure 7). Note that the VLPO regions have similar synchronization during the day, but the left hemisphere VLPO is significantly more synchronized at night. This implies that the VLPO regions, which can be classified as exhibiting a phase-cluster chimera state based on the bursting state differences shown in Figure 6B, could also be described as exhibiting a classical dynamical chimera state (in which one group is synchronized and the other is comparatively desynchronized) at night. Likewise, the right hemisphere AMIN region is significantly more synchronized than the left AMIN region during the day, again indicative of a classical chimera state. This can be illustrated more clearly, for example, for the VLPO region, by showing the synchronization indices of the right and left VLPO on the same plot (Figure 8).

**FIGURE 9:**
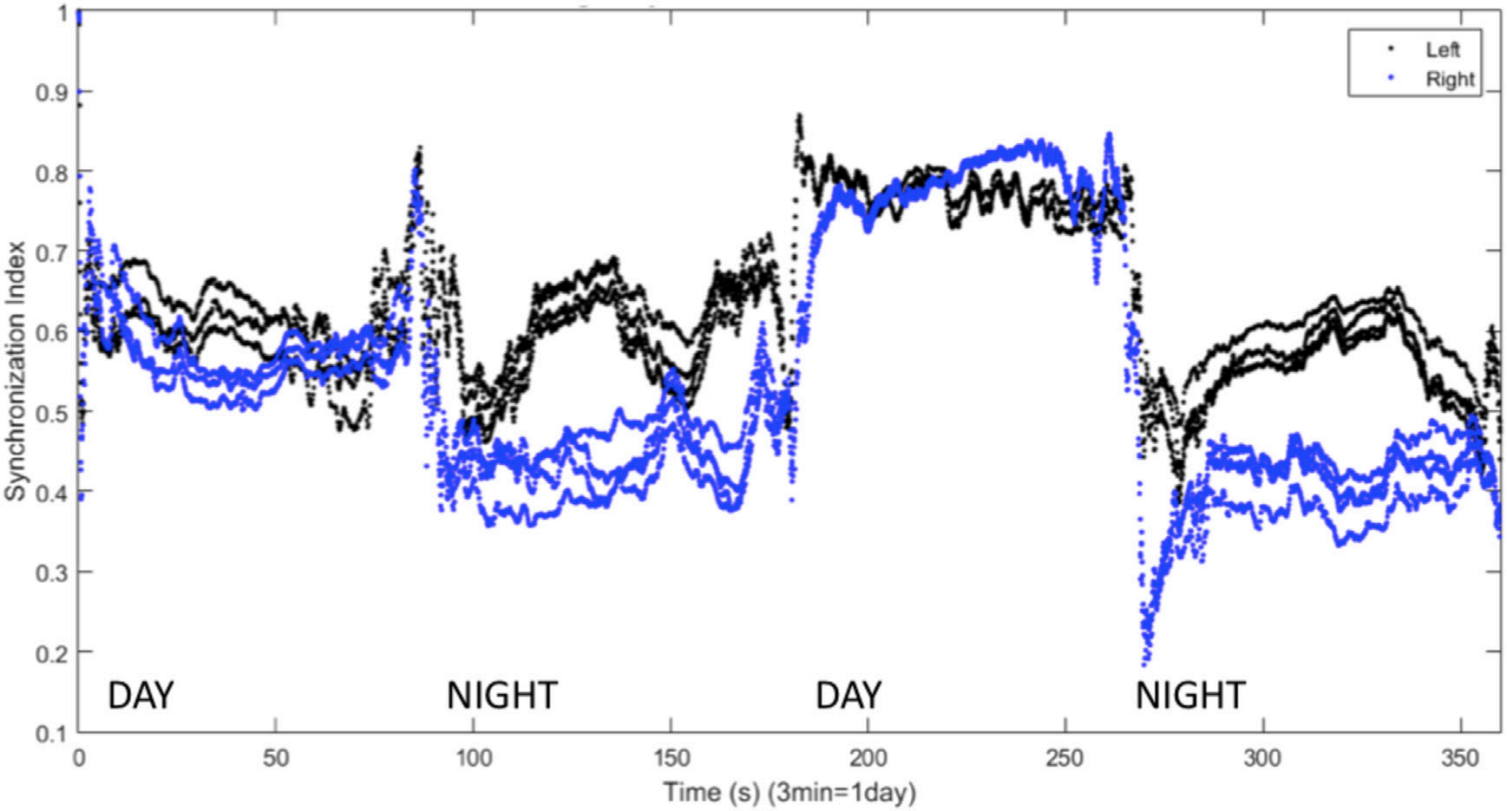
Synchronization indices calculated over a 10-spike window with a 1-spike step forward, for left (black line) and right (blue line) hemisphere VLPO, showing asymmetric sleep with inhibitory coupling between the hemispheres. Parameters are $g_{A}=g_{V}=0.000045$ , $g_{AV}=-0.0000275$ , $g_{VA}=-0.0000425,\;\;g_{CA}=0.00115$ , $g_{CV}=-0.0019$ , $g_{LR}=g_{RL}=-0.00002$ , and I=2.00 , with 3 neurons per region.

**FIGURE 10:**
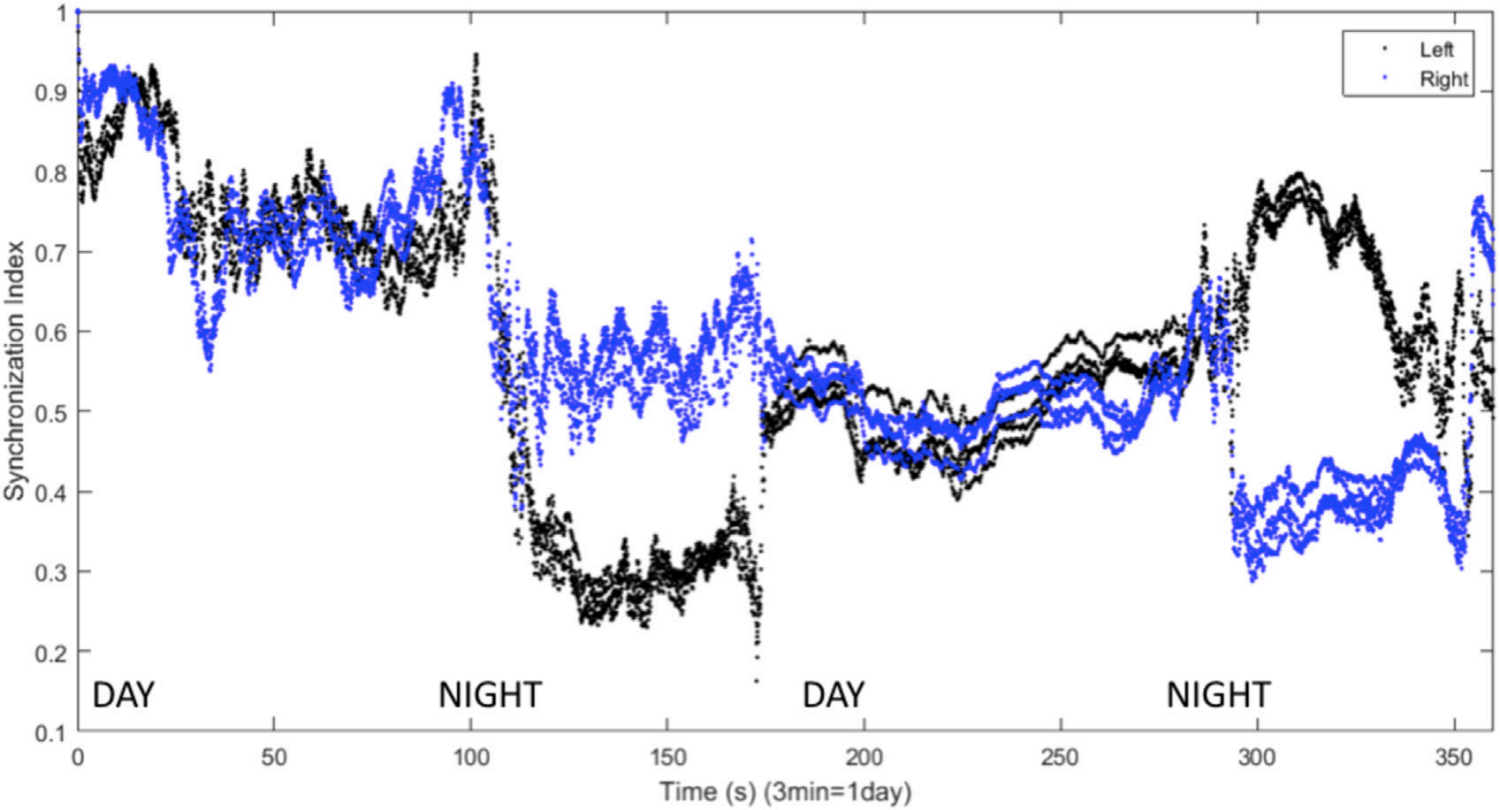
Synchronization indices calculated with a 10-spike sliding window and a 1-spike step forward for left (black line) and right (blue line) hemisphere VLPO, with inhibitory interhemispheric coupling. Note the interhemispheric switching: the right VLPO is more synchronized on the first night, and the left VLPO on the second. Parameters are $g_{A}=g_{V}=0.000045$ , $g_{AV}=-0.0000275$ , $g_{VA}=-0.0000425,\;\;g_{CA}=0.00115$ *,* $g_{CV}=-0.0019$ , $g_{LR}=g_{RL}=-0.000025$ , and I=2.25 , with 3 neurons per region.

Kedziora et al. (2012) found that inhibitory connections were necessary for the production of UHS in a computational model. Inhibitory coupling is also more likely to produce chimera states, though excitatory coupling can produce chimeras as well (Tinsley et al., 2012; Glaze et al., 2016). The results shown above all involve symmetric excitatory coupling between the hemispheres ( $g_{LR}=g_{RL}>0$ ). For symmetric inhibitory interhemispheric coupling ( $g_{LR}=g_{RL}<0$ ), chimera-like behavior is also observed. This is shown in Figure 9, where the VLPO regions exhibit significantly different degrees of synchronization on successive nights. Figure 10 also illustrates such behavior, but also shows interhemispheric switching: the left VLPO remains more synchronized during the first night, while the right is more synchronized on the second night. Other simulations with inhibitory coupling show asymmetric sleep (a wide synchronization gap between the right and left VLPO regions) punctuated by a brief collapse into symmetric BHS before a return to asymmetry, reminiscent of the shifts known to occur in patients with sleep apnea. This instance of apneic sleep demonstrates that the model is able to simulate not only UHS and asymmetric sleep, but also changes in sleep state associated with a sleep disorder.

## Discussion and Conclusions

We have presented a model of sleep dynamics based on coupled subgroups of individual Hindmarsh-Rose neurons and a circadian drive. In contrast to neuronal mass models such as that of Kedziora et al. (2012), this approach allows for the investigation of the synchronization within, as well as between, subgroups. We observe changes in synchronization within the sleep-promoting region and within the wake-promoting region as the system transitions from day to night (Figure 2). In a two-hemisphere version of the model, shown schematically in Figure 3, we find chimera-like and phase cluster states analogous to both asymmetric bihemispheric sleep (BHS), and unihemispheric sleep for both excitatory and inhibitory interhemispheric coupling (Figures 5–9). We also observe interhemispheric switching (Figure 10). These results indicate that chimera dynamics in coupled neural models can be used to model the unique dynamically asymmetric sleep states observed in a wide range of species, including human subjects suffering from pathological sleep conditions such as sleep apnea (Abeyratne et al., 2010; Rial et al., 2013) and the asymmetric sleep observed in the “first night effect” (Tamaki et al., 2016).

In Figure 2, the AMIN neurons are observed to have higher synchronization than the VLPO during the simulated night, while they are less synchronized than the VLPO during the simulated day. While this result is consistently observed in the model, the result should not be overinterpreted. A similarly structured model (Glaze, 2019) using a Hodgkin-Huxley-type neural model, the Huber-Braun model (Braun et al., 1998), shows more synchronization in AMIN than VLPO during the day and less at night, suggesting that there may be significant model-dependence in the dynamics. Single-unit recordings from sleep-promoting and wake-promoting neurons *in situ* would provide an experimental test of whether such synchronization differences exist, and studies using cultured cells on a chip could determine what neuronal properties lead to state-dependent differences in synchrony.

At the whole brain level, EEG recordings suggest that human brain activity is more synchronized during sleep (Krueger et al., 2008; de Andrés et al., 2011; Schwartz and Kilduff, 2015), but this data does not provide resolution at the level of small nuclei within the brain. The results in Figure 2 suggest the hypothesis that relative changes in neural synchrony may occur between sleep-promoting and wake-promoting nuclei during the circadian cycle. This could be investigated with single unit recordings in *in vitro* studies such as brain slice experiments, including cells from the SCN, VLPO, and locus coeruleus, as well as *in vivo* recordings. Reciprocally, further model development will be informed by experimental measurements of local synchronization dynamics *in vitro* and in the intact brain, for example single-unit recordings like those of Takahashi et al. (2010) in the locus coeruleus, and Sakai (2014) in the SCN.

Like other models of neural chimera states (Glaze et al., 2016; Santos et al., 2017; Majhi et al., 2019), the present model includes a Gaussian white noise term (Eq. 2a). While this produces instantaneous differences between the simulated hemispheres, these differences will average to zero, since the noise is applied using an identical algorithm to each neuron at each time step. The differences observed between the dynamical behavior of the two hemispheres, as shown in Figures 5, 6, moreover, are over a much greater time scale than these instantaneous fluctuations, which occur on the time scale of the integration time step. Chimera states have been found to be robust to the presence of noise (Laing, 2012; Panaggio and Abrams, 2015; Loos et al., 2016; Bukh et al., 2018); tuning the noise amplitude has been shown to affect the lifetime of chimera states (Zakharova et al., 2016), and a coherence resonance effect has been observed in which an intermediate amount of noise enhances the occurrence of chimera states (Semenova et al., 2016; Zakharova et al., 2017; Tang et al., 2019; Wang and Liu, 2020).

The model described here could be further developed with the addition of realistic features other than noise. For example, the circadian drive could be decoupled into an intrinsic SCN rhythm and an external drive, in order to examine the effects of circadian misalignment (Fischer et al., 2016), jet lag (Sack et al., 2007a; Sack et al., 2007b) and drugs such as caffeine (Puckeridge et al*.*, 2011) or other non-photic stimuli (St. Hilaire et al., 2007).

An additional wake-promoting region, such as orexinergic (ORX) neurons from the lateral hypothalamic area (LHA) could shift the dynamics of the model. These neurons release the neurotransmitter orexin (also called hypocretin), a crucial element of sleep-wake regulation. Lack of orexin can cause narcolepsy (Sakurai, 2007; Schwartz and Kilduff, 2015). ORX is present in many models of sleep, including the UHS model developed by Kedziora et al. (2012) and the sleep/wake flip-flop model of Rempe et al. (2010). The ORX neurons of LHA interact with both VLPO and AMIN (Saper and Lowell, 2014), and could strengthen and stabilize the wake state, as well as provide additional factors regulating the emergence of chimera-like states.

In conjunction with the circadian drive, the homeostatic drive builds up sleep pressure as time spent awake accumulates, and decreases sleep pressure with time spent asleep. This relationship was put forward by Borbély (1982) and modeled by Daan et al. (1984). The homeostatic drive has been proposed to be regulated by neurons in the VLPO and median preoptic nucleus (MnPO) (Gvilia et al., 2006), as well as by ORX (Postnova et al., 2009). Addition of a homeostatic drive term to the present model would also allow the investigation of how processes such as sleep debt (Borbély et al., 2016) would affect sleep asymmetry.

The effects of other regions involved in sleep regulation such as the MnPO could also be investigated. Located in the hypothalamus, this region promotes the transition from wake to sleep (Gvilia et al., 2006). Firing ahead of the switch to sleep, MnPO may add to sleep pressure (Saper et al., 2010). It also inhibits the LHA, promoting the wake-to-sleep transition (Suntsova et al., 2007), in opposition to the effects of ORX. Another key region in sleep regulation is the extended ventrolateral preoptic nucleus (eVLPO). This region inhibits the REM-off regions in the brain, allowing the transition from NREM to REM sleep (Lu et al., 2006; Rempe et al., 2010). The eVLPO exists in a flip-flop switch with both AMIN neurons (which inhibit REM-on regions) and the VLPO (to regulate the switching between NREM and REM sleep) (Rempe et al., 2010). This region makes inhibitory projections onto the LC, where the AMIN neurons reside (Saper et al., 2010). However, note that REM sleep does not occur during UHS (Rattenborg et al., 2000), and REM is largely, if not entirely, absent in aquatic mammals (Madan and Jha 2012; Lyamin et al., 2018).

In the present model, the VLPO and AMIN regions have been modeled with identical Hindmarsh-Rose neurons. The difference between the two regions was implemented only *via* the differential input from the circadian drive. In a more realistic model, parameters could be used which would better reflect the firing patterns typical of these regions, as these become better understood from *in vitro* studies and single unit recordings; more realistic neural models, of course, could be used as well, though this would come with the inevitable tradeoffs of increased computational time and additional parameters. Size effects could also play a role as the number of neurons in each region is increased, though preliminary results suggest that size effects have minimal effect on AMIN and VLPO synchronization in a one-hemisphere model using Huber-Braun neurons (Glaze, 2019).

Another important direction for investigation is the use of connectivity between regions based on empirical data (Wang and Liu, 2020). A recent study by Ramlow et al. (2019) found partial synchronization in a network of FitzHugh-Nagumo oscillators with connections based on empirical data from healthy human subjects. They observed asymmetries in the synchronization dynamics analogous to unihemispheric sleep, but found that these were due to the structural asymmetry in the model rather than to a true chimera effect. Santos et al. (2017) took a similar approach in developing a network model of the cat cerebral cortex, using the Hindmarsh-Rose equations, based on an empirical connectivity matrix. Such studies drive home the importance of combining both fundamental dynamical studies and empirical data by introducing different time delays or asymmetric coupling matrices within and across hemispheres.

Given that real brains exhibit structural asymmetry, does the chimera-state approach provide a reasonable model for unihemispheric sleep? Structural asymmetry has been shown to be important in driving the dynamics of the default mode network, whch is active during a state of quiet (awake) resting (Saenger et al., 2012). Recent advances in understanding how the brain’s structure shapes its dynamics (Deco et al., 2011; Shen et al., 2015), as well as advances in the understanding of the neural connectome (Sporns et al., 2005; Kaiser, 2017; Betzel and Bassett, 2018) can provide the basis for models incorporating realistic structural asymmetries. This structural information could be combined with the modeling of individual neural oscillators, in order to investigate local synchronization changes, not only in bespoke code, but also in simulation platforms such as NEST (Kunkel and Schenck, 2017).

Incorporation of more realistic connectome data will be an important future step in determining the balance between dynamics and structural connectivity in driving sleep dynamics and other possible chimera-like states in the brain. Of particular interest in this regard will be studies such as Petkoski et al. (2018) and Petkoski and Jirsa (2019) which highlight the role of time delays and phase lags in large-scale brain network synchronization, because time delays are an important component of chimera dynamics (Tinsley et al., 2012).

Despite its obvious importance, structural asymmetry alone is unlikely to be the primary driver of sleep dynamics in species which exhibit hemispheric switching during unihemispheric sleep. Such switching must be, at its core, dynamically driven, since structural architecture is surely not rewired multiple times each night. Expanded versions of the chimera-generating model described here, with an emphasis on local synchronization within neural clusters, will, in combination with experimental data, be essential for decoupling dynamically-driven sleep asymmetries from those determined by functional architecture.

## Data Availability

The raw data supporting the conclusion of this article will be made available by the authors, without undue reservation.
